# Research on the detection model of mental illness of online forum users based on convolutional network

**DOI:** 10.1186/s40359-023-01460-4

**Published:** 2023-12-04

**Authors:** Yuliang Guo, Zheng Zhang, Xuejun Xu

**Affiliations:** 1 Las Positas College Dublin High School, Livermore, 94551 USA; 2 School of Computer and Software, Nanyang Institute of Technology, Nanyang, 473004 China; 3 College of Education, Hubei University Wuhan University of Arts and Science, Wuhan, 430062 China

**Keywords:** Social media, Mental Illness, Depression detection, Neural network, Feature representation

## Abstract

Recently, there will be more than 4.62 billion social media users worldwide. A large number of users tend to publish personal emotional dynamics or express opinions on social media. These massive user data provide data support for the development of mental illness detection research and have achieved good results. However, it is difficult for current mental illness detection models to accurately identify key emotional features from a large number of posts issued by users to detect problem users. In view of the fact that the existing models cannot more accurately extract the words with high emotional contribution in the content of user posts, this paper proposes two hierarchical user post feature representation models, named Single-Gated LeakReLU-CNN (SGL-CNN) and Multi-Gated LeakyReLU-CNN (MGL-CNN). We leverage these 2 models to identify users with mental illness in online forums. For all posts published by each user within a certain time span, the model proposed in this paper can identify key emotional features in them and filter out other unimportant information as much as possible. In addition, the addition of gating units in this paper can significantly improve the performance of emotion detection tasks. The experimental results based on the task of RSDD dataset prove that the performance of the model proposed in this paper is superior to that of the existing methods.

## Introduction

The development of the times and social changes have made social competition more and more fierce, making people face various pressures in life, study, emotion and employment. When faced with stress, people's psychological state is prone to change, resulting in various psychological abnormalities. If psychological abnormalities are not adjusted in time, it will cause serious emotional disorders. Depression is one of the typical and common mood disorders. Depression has received much attention in the field of mental health. According to available research, approximately 280 million people suffer from depression worldwide, and 5.0% of adults suffer from depression [1]. Among many mental illnesses, depression is the most common one, the second most common disease that plagues global public health problems, and the main cause of the total global disease burden. After the COVID-19 epidemic, the global burden of mental disorders has become heavier. The cases of major depressive disorder and anxiety disorder have increased by 28% and 26% respectively, and the number of patients with depression has surged by 53 million, an increase of 27.6% [2, 3]. According to the World Health Organization, the global incidence of depression is about 11%, and it is estimated that by 20,230, depression will become the main cause of disability worldwide [4]. Literature [5] conducted a cross-sectional epidemiological survey on the prevalence of mental disorders at 157 monitoring points in 31 provinces in China. The results showed that in the past 30 years, the lifetime prevalence of depression in China was 6.8%, the prevalence rate during the year was 3.6%. Suicidal ideation is one of the main symptoms in a depression diagnosis. About 2/3 of depressed patients have had suicidal ideation, and about 25% of depressed patients have had suicidal behavior. In China, 287,000 people die by suicide every year, 63% of the suicides have mental disorders, and 40% suffer from depression [6]. Depression not only causes great harm to oneself, but also imposes a heavy economic burden on society. The direct and indirect economic losses caused by depression in China are as high as 80 billion dollars per year [7].

At present, most researches on the detection of mental illness focus on depression. As social media continues to grow, more and more users suffering from mental illness are turning to online forums to express their mental health concerns and seek help and treatment. For example, online support forums like Reddit have many self-reported depression patients, and they provide a lot of negative emotional information for scholars to study [8]. Traditional mental illness detection usually requires patients to seek help from a psychologist on their own initiative. However, considering that the development of mental health services in remote and underdeveloped areas is not perfect and the cost of diagnosis is relatively high, many people cannot get timely diagnosis and miss the best treatment period. Therefore, automatic diagnosis and identification of depressed patients based on human emotions has become the key to the prevention and treatment of depression.

With the gradual popularity of social media and the continuous development of computer technology, the number of users joining social networks is on the rise. More and more netizens are inclined to express their opinions and emotional dynamics in social media, and more and more personal posting information can be collected on the Internet. Social media has gradually become an important way for the public to share the latest emotional information and discuss hot spots of public opinion and an effective channel for obtaining information. On social media, mental health issues have gradually become a hot topic in the field of health communication. How to dig out the potential medical value and useful information from these massive personal data, and at the same time devote enough attention and appropriate medical auxiliary treatment is a very challenging and socially significant research. The use of social media data for depression detection can maximize the convenience of big data, and can also more effectively identify some patients with potential mental illness problems. This group of people may not be diagnosed but still have a high risk of disease.

The reason why social media can attract a large number of users to use and express their true inner feelings is mainly reflected in two aspects. First, social media provides users with a relatively closed and safe environment. Due to the anonymity of social media, people are more willing to express their opinions in the online world. Because users can lose their defenses and burdens in an anonymous environment, the cost of speaking is significantly reduced. Second, due to the virtuality and invisibility of the Internet, users do not have to deliberately maintain their real images, and can express their true inner feelings even more carelessly. Therefore, using specific sentiment analysis algorithms to process feature words with strong negative semantics posted by users on social media, users with potential psychological problems can be discovered as early as possible. This can help those potential patients to intervene in advance, so as to avoid the aggravation of psychological problems and the occurrence of inactive medical treatment [9]. In addition, personal language has a very important impact on the disclosure of mental illness [10]. During psychotherapy, doctors can make a diagnosis through the patient's language, so as to quickly diagnose mental diseases including depression, anorexia, and autism. Therefore, it is very necessary to use language to explore deeper problems of mental health. As the main carrier of individual information, social media is more worthy of researchers to explore and mine rich user language information, so as to better assist in the diagnosis of some potential mental illness patients.

The research in this paper is mainly based on a large amount of user data generated in social media. The main purpose is to accurately identify users with mental illness in a large amount of social media data. The main limitation of existing methods for mental illness detection is that some detection models are mostly based on machine learning algorithms, which require a lot of effort in feature engineering. However, the deep learning-based model does not pay much attention to the improvement and optimization of the model, and it is difficult to accurately identify key emotional features from a large number of posts published by users to detect problem users. In addition, due to the long time span of users posting, existing models do not take into account the time correlation and dependence between posts. These problems make the existing detection models still have a lot of room for improvement in terms of performance.

This paper adopts a layered structure to simulate the posting process of users, introduces the concept of layering and gating weight into the field of mental disease detection, and proposes two layered neural network models for mental disease detection, named SGL-CNN respectively and MGL-CNN. User datasets from different social media all contain a certain number of posts. For all posts published by each user within a certain time span, the two models proposed in this paper can be used to identify the real key emotional features contained in them, and suppress other unimportant feature information as much as possible.

### Related work

#### sentiment analysis method based on social media data

**Fig. 1:**
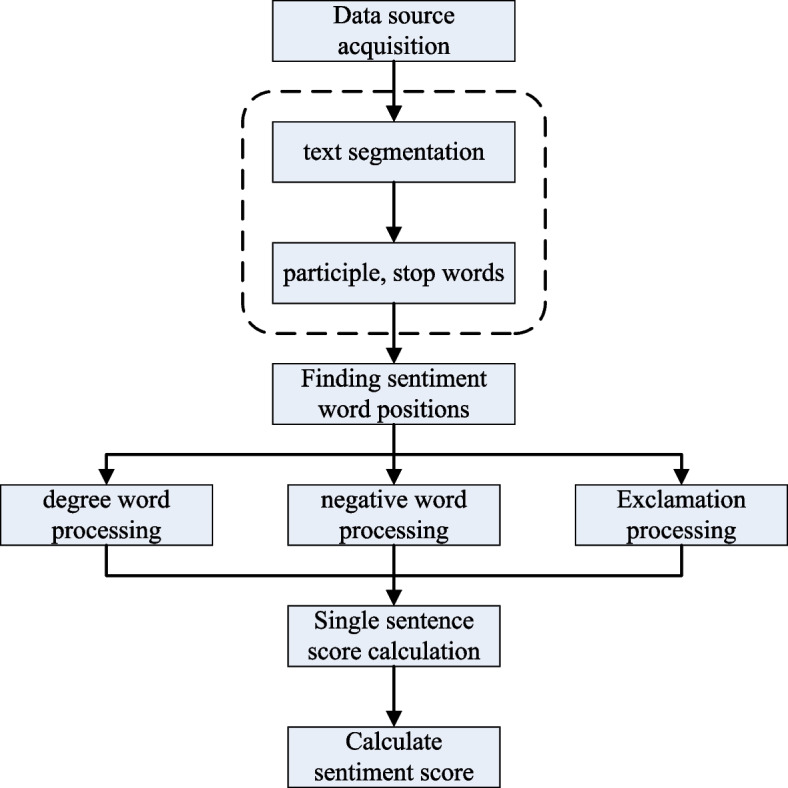
Flow chart of text sentiment analysis based on sentiment lexicon

The general sentiment analysis flow chart is shown in Fig. 1. The method based on the sentiment lexicon regards the sentiment words as an important basis for judging the sentiment polarity of the text. A large amount of manual experience and rules are needed to summarize and give certain weights to commonly used emotional words when constructing an emotional dictionary. Its general process is: first of all, it is necessary to identify the emotional words that can express the emotional tendency of the text, and a perfect emotional dictionary will be used here. Afterwards, the text is scored according to the corresponding algorithm to obtain its emotional tendency value.

Sentiment dictionaries play a very important role in sentiment analysis tasks. Relying on a large number of emotional words in the sentiment lexicon to analyze text is the key to analyzing sentiment. Sentiment dictionaries can usually be constructed manually [11], or through heuristic algorithms [12] or related algorithm construction for machine learning. The advantage of the method based on emotional lexicon is that it can manually collect professional field dictionaries and write more accurate and better quality emotional lexicons. The disadvantage is that it consumes a lot of labor.

**Fig. 2:**
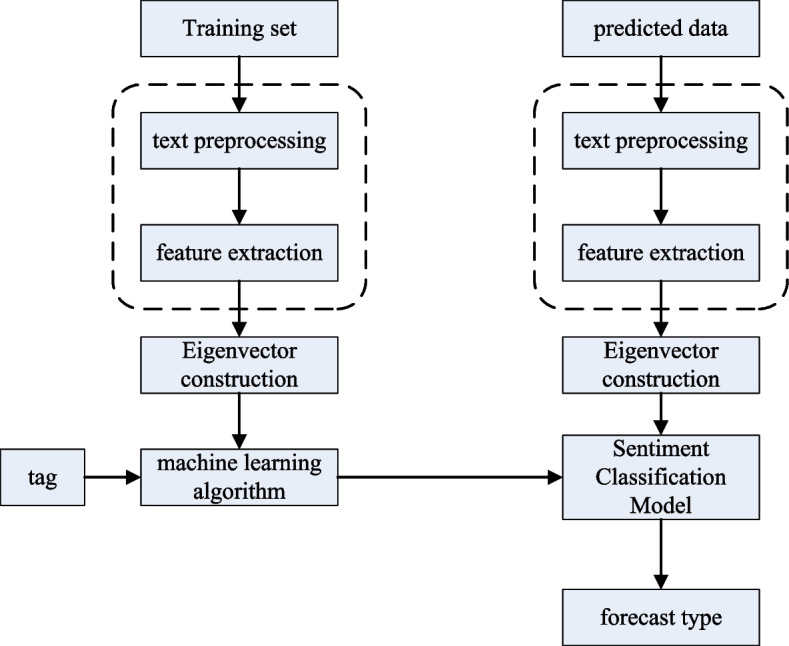
Flow chart of text sentiment analysis based on machine learning

The overall process based on the machine learning method is shown in Fig. 2. Traditional text-level sentiment analysis usually uses a combination of machine learning and feature engineering. Features are information extracted from data that is useful for outcome prediction. Sentiment classification methods based on machine learning mostly use classical classification models such as support vector machines, naive Bayesian, and maximum entropy models. The performance of most of the classification models depends on the quality of the labeled data set, and obtaining high-quality labeled data requires a lot of labor costs. Pang [7] et al. studied the effectiveness of applying traditional machine learning models (maximum entropy model, naive Bayesian and support vector machines) to sentiment classification problems and compared them with traditional topic models. Compared with the previous method, the method in [13] improves the accuracy rate by 10 percentage points. However, the method in [13] cannot express the structural features of documents, ignoring the semantic relationship between sentences. Secondly, the training and testing of the experiment are carried out on the same data set, and the trained model is highly dependent on the data set and does not have wide applicability. Turney [14] et al. extracted sentiment words from syntactic patterns, and trained machine learning models to identify the sentiment polarity of documents. Wang [15] et al. used n-grams of words as features, and chose Naive Bayes and Support Vector Machine (SVM) and its variants to implement sentiment analysis. In the paper, the authors propose a model variant of SVM using the log count ratio as the eigenvalue.

**Fig. 3:**
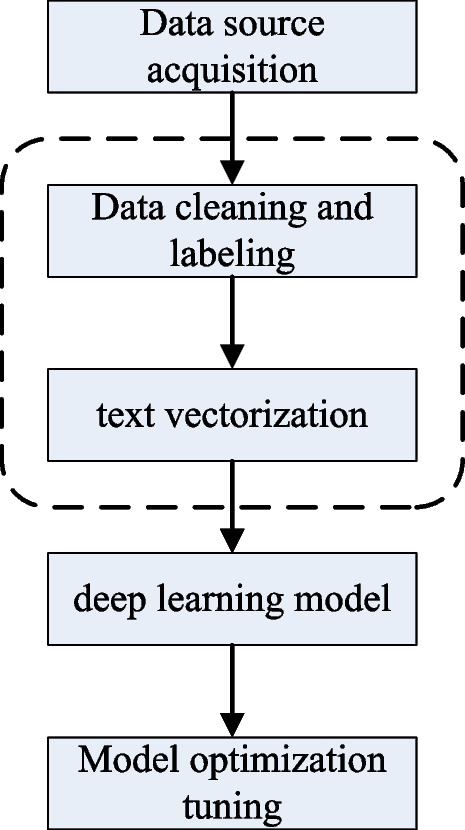
Flow chart of text sentiment analysis based on deep learning

Traditional sentiment analysis methods mainly rely on the design of feature functions (feature engineering), while deep learning allows a trainable neural network structure to represent a higher-level feature, which can greatly simplify the process of feature engineering. The process of text sentiment analysis based on deep learning is shown in Fig. 3. Part of the text classification research of deep learning focuses on the research based on the word embedding model. The other part is building and optimizing neural networks or classifiers. Two representative network models are convolutional neural network and recurrent neural network based on long short-term memory (LTSM). The convolutional neural network aims to learn to extract the hierarchical structure of key text elements, which is simple and efficient and can achieve better accuracy. Kim [16] first applied the convolutional neural network to text classification, and proposed four related variant models to achieve pre-training and word embedding performance improvement. Gated Convolutional Neural Networks [17] first introduced gated units into CNN language modeling. This model provides a linear path for the gradient while maintaining the non-linear ability to reduce vanishing gradients. Yang [18] et al. proposed a novel text classification model based on gating mechanism. The model generates a variety of gate weights through convolution kernels of different sizes to control how much important information in the text is retained. Although CNNs were originally used in computer vision, they have been very successful in NLP tasks. Because they do not rely on time series, they are easily parallelized during training, saving training time considerably. The disadvantage of CNN is that it cannot guarantee the sequential characteristics of words and sentences. Long short-term memory neural network (LSTM) can effectively alleviate the problem of gradient explosion or gradient disappearance, and learn longer dependent information. Xu et al. [19] proposed CLSTM to capture emotional semantic information of longer sequences. It adds a caching mechanism to the LSTM, and divides the memory units of the hidden layer into several groups according to the different forgetting rates, so that the group with a high forgetting rate acts as a cache. Duyu Tang [20] used a neural network with a gate mechanism to model chapters. First, a convolutional neural network was used to model the chapters in a fine-grained manner, and then a neural network with a gate mechanism was used. Zichao Yang et al. [21] proposed a hierarchical attention network for document classification. The model has a two-level attention mechanism, which is suitable for sentences and documents, and can selectively focus on information-rich words and sentences.

### Mental illness detection method based on social media data

Self-expression and social support can help improve the mental health of people with mental illness. In addition, the language users use in social media can reveal their true inner thoughts. Natural language is related to human personality, psychological state and situational fluctuations. Therefore, how to identify the language style of individuals with a tendency to depression is particularly important.

More and more depressed patients turn to online media resources (Twitter, Weibo, Reddit, etc.) to express their psychological problems and seek help [22]. Many users especially gravitate toward online web forums where they can choose to remain anonymous or remain a guest. Early detection of depression using social media data has become an effective means.

Sentiment analysis detection tasks related to mental health are similar to traditional sentiment analysis classification tasks, and some of these studies mainly use traditional machine learning methods. For example, Schwartz et al. used Facebook data to build a regression model to predict individual depression levels from multiple granularities [23]. Thompson et al. constructed a mental illness detection model based on a random forest classifier and a bag-of-words model using patient clinical records and online social media data [24]. They used the model to examine suicide risk and mental health in service members and veterans. Moreno et al. [25] used a large amount of data collected from Facebook to conduct learning and analysis of depression detection algorithms, and finally refer to the symptoms of depression patients to identify depressed users.

In addition to the good results achieved by machine learning in the exploration of depression detection, many deep learning methods have also achieved good results in text classification and sentiment analysis. These deep learning methods only rely on the content of the text itself, not on any other external features. Gui et al. [26] proposed a novel collaborative multi-agent model to detect depressed groups in the Twitter dataset. The model includes a text feature extractor and image feature extractor. The text feature extractor employs a gated recurrent unit and a convolutional neural network to extract the sentiment features of the dataset. Yang et al. [27] built two effective target-related emotion classification models using bidirectional long-term and short-term neural networks. In addition to long-short-term neural networks and recurrent neural networks, convolutional neural networks (CNNs) are also actively used in text classification in the medical field. A general depression detection model based on convolutional neural networks was proposed by Nemeth et al. They used it to combine users' posts with user language, posting characteristics to assess users' depression and self-harm risks [28]. Subsequently, Cong et al. proposed a deep learning-based method to solve the depression dataset RSDD with imbalanced positive and negative data volume [29]. Liang et al. introduced an emotional feature extraction model based on graph convolutional neural network [30]. The model can selectively output sentiment features according to a given aspect or entity.

### Mental illness detection model based on gating weights and convolutional networks

**Fig. 4:**
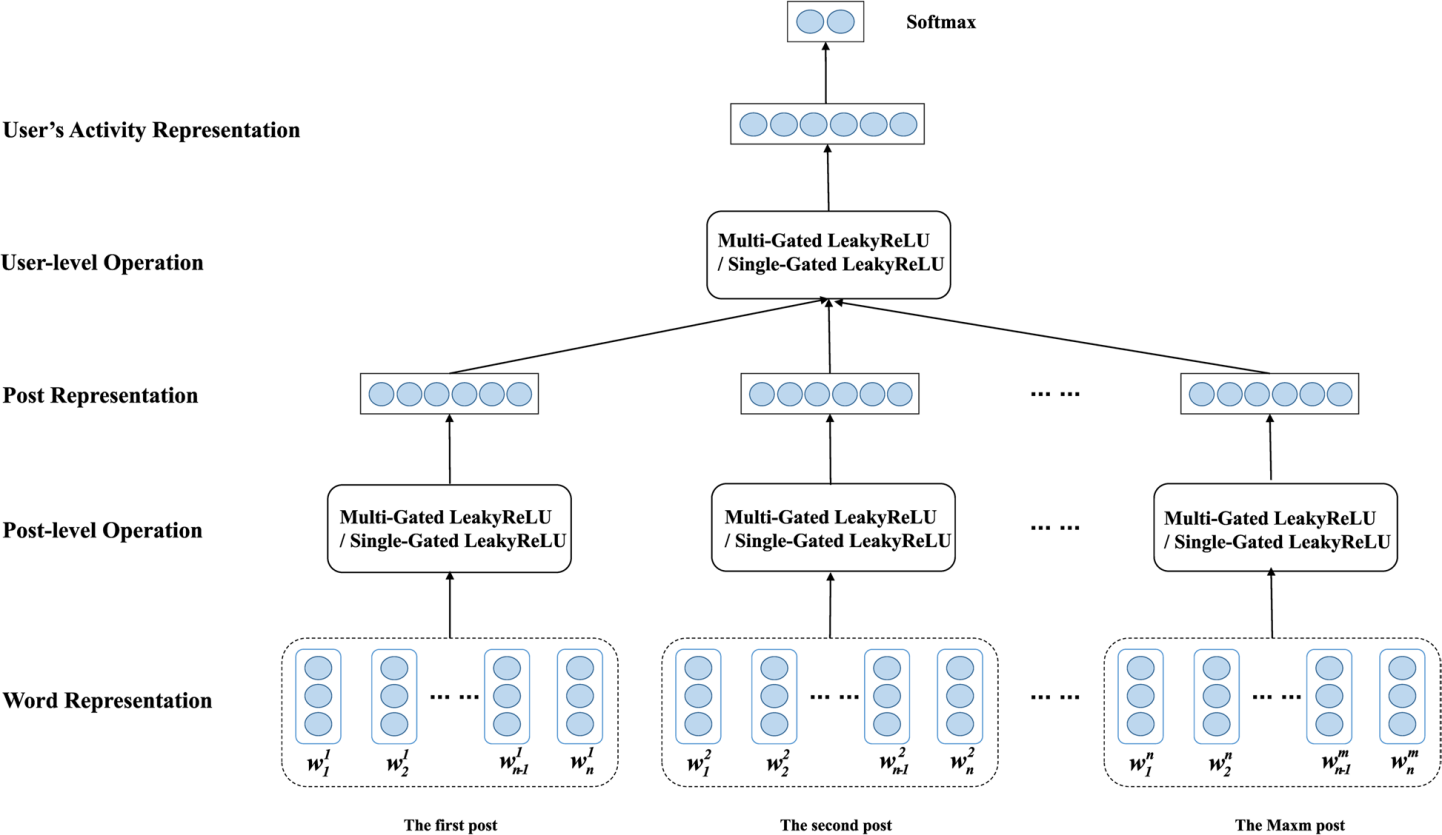
The overall block diagram of the mental illness detection model proposed in this paper

This paper introduces two novel hierarchical mental illness detection models to identify mental illness patients in web forums, which are named MGL-CNN and SGL-CNN. Since the user's overall data consists of a list of posts, and each post is composed of a list of words, our model can be split into 2 parts. One part is the post feature representation layer, and the other part is the user's overall activity representation layer. The basic principles of the two models proposed in this paper are as follows. The model first produces a continuous post feature representation from the word representations in the post. The model then takes the post feature representation as input to the second part to obtain the overall emotional state representation of the user. Finally, the model outputs the user's overall emotional activity representation as a feature output for mental illness classification. The overall block diagram of the mental illness detection model described in this chapter is shown in Fig. 4. The two models of MGL-CNN and SGL-CNN have many similarities, and the difference is mainly in the number of gating units.

Previous natural language processing methods require more training time and computational overhead because most of them adopt long-short-term memory and attention mechanism models to predict emotional polarity. The model proposed in this paper can solve this problem well. First, we replace the idiomatic recurrent connections in recurrent networks with gated temporal convolutions. Second, we use a special convolutional encoder to convolve the input and obtain gating weights independently. The advantage of this is that patients with a tendency to mental illness can be more accurately identified. Compared with previous methods, the method proposed in this paper has no time dependence, and can easily perform parallel operations in user documents, thereby improving computational efficiency.

**Fig. 5:**
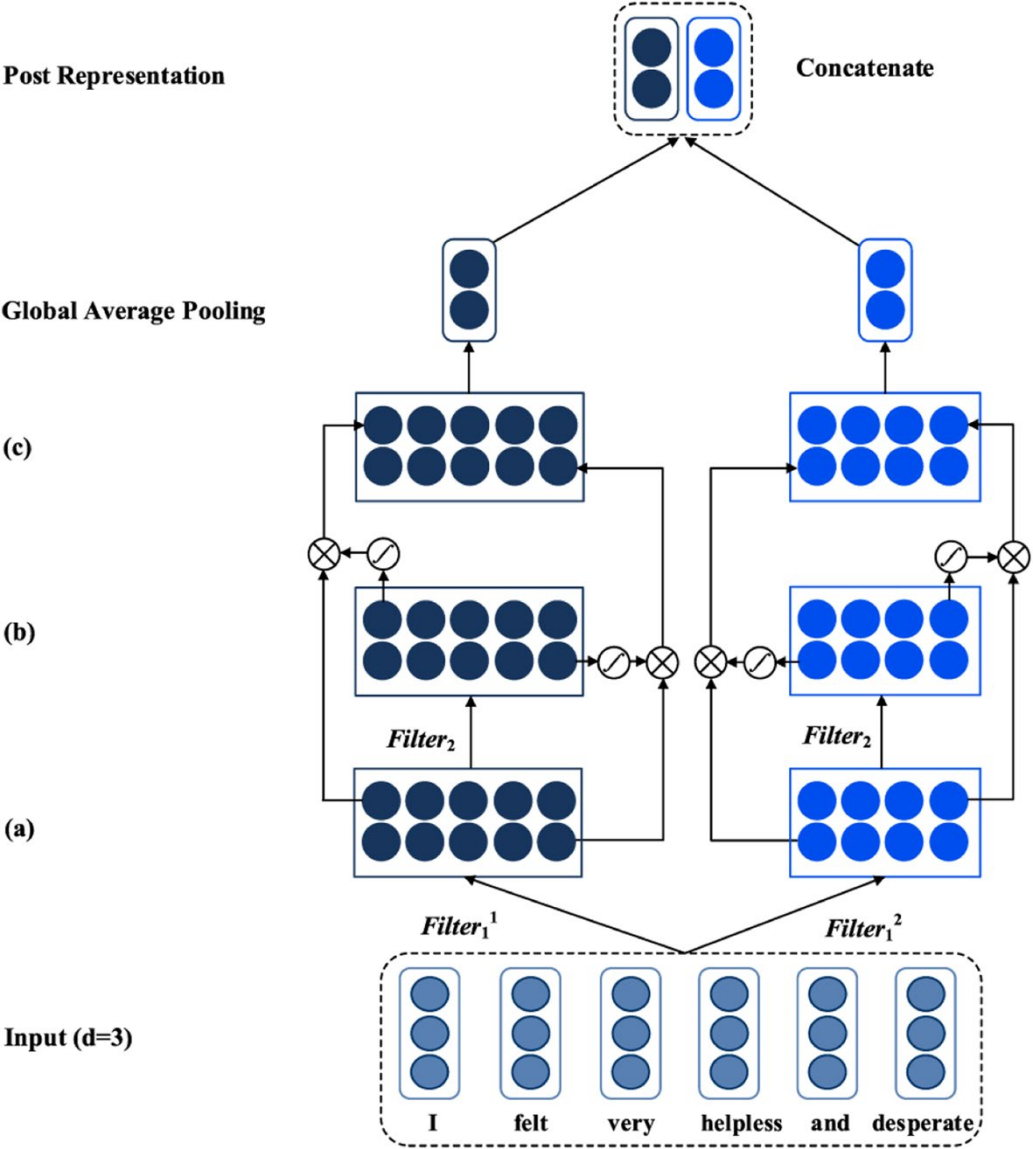
The specific block diagram of the post feature representation layer in the MGL-CNN model

**Fig. 6:**
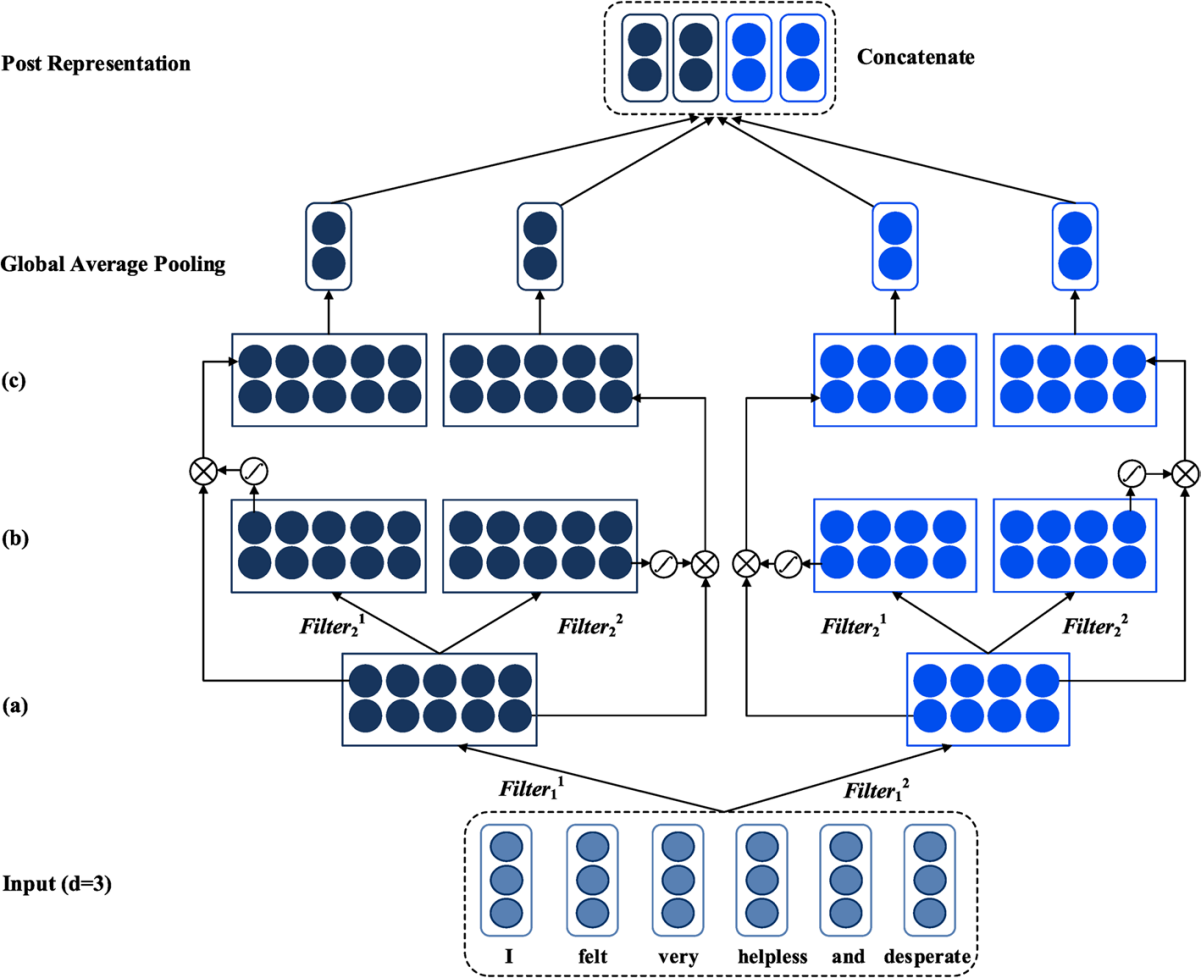
The specific block diagram of the post feature representation layer in the SGL-CNN model

In the model proposed in this paper, the structure of the user activity representation layer is the same as that of the post feature representation layer. The input of the model can be passed by the multi-layer convolutional neural network of the gating unit, so that the limited context information can be fully utilized to obtain the key features of the post representation with maximum efficiency. Figure 5 depicts the specific details of the post feature representation layer of SGL-CNN. The post feature representation layer of MGL-CNN is shown in Fig. 6. It is worth noting that both SGL-CNN and MGL-CNN are composed of 2 convolutional layers and a global average pooling layer. The main difference between them is the number of gating weights generated. We briefly explain here, taking the post representation layer in MGL-CNN as an example, its first convolutional layer first obtains the abstract feature map. The acquisition process of this feature map uses two convolution kernels of different sizes. Next, different gating weights are obtained by configuring the second convolutional layer of the gating unit. Then, the abstract feature map derived by the first convolutional layer is multiplied element-wise by the gating weights derived by the second convolutional layer to obtain the feature representation of the post.

For ease of understanding, we next mainly describe the process of extracting a feature from a filter. Each word is represented by a vector stored in the word embedding matrix. We denote each post by a user as {w1, w2,…,wi,…wn}.We let xiϵRd be the d-dimensional word vector corresponding to the i-th word in the post. A post vector consisting of n words can be formulated as follows:

1 $${X}_{1:n}=\left[{x}_{1},{x}_{2},\dots ,{x}_{n}\right]$$

The representation of a single post in the first convolutional layer can be done by a CNN and multiple convolutional filters of different widths, for which we refer to [29]. The advantage of this is that convolution filters of different widths can be considered as feature extractors. In this way, multi-granularity local information can be obtained, such as N-Grams. As an example, a convolutional filter of width 2 can actually capture the semantics of Bi-Grams in user posts. Multiple feature maps can be obtained by multiple convolution filters of different sizes. In order to generate a new feature, we can apply a convolution kernel KϵRs with a stride of 1 and size s to a window of s words. We use Xi:i + s-1 to represent a concatenation of word vectors under a fixed window of s, through which we can generate a new abstract feature αi.

2 $${\alpha }_{i}=\left(K*{\mathrm{X}}_{i:i+s-1}+b\right)$$

where bϵR is a bias term, * represents a convolution operation, and f is an activation function. In this section, we use LeakyReLU. We apply it to posts, and we can generate a feature map A, which is expressed as follows:

3 $$A=\left[{\alpha }_{1},{\alpha }_{2},\dots ,{\alpha }_{n-s+1}\right]$$

where AϵR^(n−s+1)×1^. Then, each feature map produced by filters of different sizes is output to the second convolutional layer. The second convolution layer includes a convolution kernel and a gating unit. The goal of the second convolutional layer is to derive differentiated gating weights to better extract feature information from the first convolutional layer. We make the convolution kernel FϵR^h×1^, which is used to obtain the context feature A. The convolution kernel F acts on the feature al to obtain the gating weight.

4 $${g}_{l}\left\{\frac{f\left(F*{\mathrm{A}}_{l-\frac{b}{2}:l+\frac{b}{2}-1}+b\right)\left(\mathrm{h} \mathrm\,{is} \mathrm\,{an\, even\, number} \right)}{f\left(F*{\mathrm{A}}_{l-\frac{b-1}{2}:l+\frac{b-1}{2}+b}\right)\left(\mathrm\,{h\, is\, an\, odd \,number}\right)}\right.$$

The gating weights generated by the convolution of the feature map A and the convolution kernel F can further generate a gating weight matrix.

5 $$G=\left\lfloor{\mathrm g}_1,{\mathrm g}_2,\dots,{\mathrm g}_{n-s+1}\right\rfloor$$

Assuming that the number of convolution kernels in the second convolutional layer is m, we can extract different gating weight matrices from the gating unit of MGL-CNN. Then, the output feature map O can be further obtained through the gating weight matrix, which can be expressed as:

6 $$O=A\otimes \mathrm{G}$$

where ⊗ means that the elements between the matrices are multiplied.

The output O in the process of modeling from words to sentences is conditioned by gating weights G. These gating weights are similar to the attention weights learned in the attention mechanism, which can more accurately assign different importance to each word, which is conducive to improving the performance of the model. These gating weights are multiplied by the feature map A to control which information should be propagated through these layers.

To obtain the global information of a post, we feed the output of the second convolutional layer into a global average pooling layer, and concatenate all output features to obtain the final representation of a single post. To compute a user's overall psychologically acquired representation, the preordered post representations are fed into the user activity representation layer. Subsequently, the user's features are passed to a fully connected softmax layer whose output is a probability distribution over the labels. The loss function of this model uses the cross entropy loss function. Assuming that the target sentiment distribution of each document can be represented by pT, then the loss *C* value can be calculated as:

7 $$loss=-\sum_{i\epsilon T}\sum_{j=1}{p}_{j}^{T}\left(i\right)\cdot log\left({p}_{j}\left(i\right)\right)$$

In formula (7), T represents the data used for training, and C represents the number of experimental categories.

### Experimental results and analysis

This section mainly conducts experimental tests on the two mental disease detection models of MGL-CNN and SGL-CNN mentioned above to verify their effectiveness and accuracy. This chapter mainly selects three large-scale data sets about mental illness in social media. One is the Reddit Self-Reported Depression Diagnosis (RSDD) dataset, the other is the Early Detection Dataset of Depression (eRisk2017), and the third is the Anorexia Dataset (eRisk2018).

### Experimental dataset

The latest large-scale depression detection dataset, the Reddi Self-Reported Depression Diagnosis (RSDD) dataset contains more than 9,000 users diagnosed with depression, and about 107,000 control users with healthy mental status. Non-depressed users were selected by matching candidate non-depressed users with diagnosed users. Data related to mental illness status in social media data usually has strong privacy and sensitivity. Therefore, the user's personal data risks and privacy issues must be considered when obtaining data. The RSDD dataset used in this paper only includes Reddit posts that users actively publish.

The authority of this data set is mainly reflected in the user selection. The users marked as depressed mainly consist of the following two points. The first point is that the patient wrote some self-diagnosed depression sentences many times in the post. Use these high-precision sensitive sentences to determine the diagnostic user group. The second is that some users who do not meet the data set construction rules will be excluded. These rules mainly include that the number of user posts is less than 100, and the number of depression-related characters mentioned in a single post does not exceed 80. Table 1 lists the specific user statistics of the RSDD dataset.

**Table 1:** RSDD dataset user statistics

dataset	diagnosed with depression	general user
training	3070	35,753
test	3070	35,775
validation	3070	35,746
Total number of data	9210	107,274

The early detection data set of depression (eRisk2017) is mainly used to carry out the early risk detection task of depression. The relevant data statistics of this data set are shown in Table 2.

**Table 2:** eRisk2017 depression data user statistics

	test		validation	
	depressed user	control user	depressed user	control user
User number	83	403	52	349
total number of posts	30,851	264,172	18,706	217,665
Average number of posts per user	371.7	655.5	359.7	623.7
Average number of words in a single post	27.6	21.3	26.9	22.5

An early detection dataset for anorexia (eRisk2018) is similar to an early detection dataset for depression. It was mainly used for exploratory tasks on early risk detection of anorexia. The dataset has a small number of users, mainly consisting of 61 anorexia patients and 411 mental health users. Table 3 shows the statistics of the eRisk2018 anorexia dataset training set and test set. Although there are not many users in this data set, each user has a long history of posting (the average number of posts per user exceeds 300, and the number of words contained in each post exceeds 20), so a single user The amount of data is huge.

**Table 3:** eRisk2018 anorexia dataset user statistics

	test		validation	
	anorexic user	control user	anorexic user	control user
User number	20	132	41	279
total number of posts	7452	77,514	17,422	151,364
Average number of posts per user	372.6	587.2	798.9	670.6
Average number of words in a single post	41.2	20.9	35.7	20.9

### Experimental evaluation method

The detection and analysis of mental health problems is essentially a text classification or multi-classification problem, so this paper uses precision (Precision), recall (Recall), and comprehensive evaluation indicators (F-Measure) to evaluate the results of emotional detection. The precision rate refers to the ratio of samples predicted as positive emotions (negative emotions) to real positive emotion samples in the results of emotion prediction. The precision calculation formula is as follows:

8 $$precision=\frac{TP}{TP+FP}$$

Recall refers to the ratio of positive samples in a sample that are correctly estimated. There will also be two situations, one is to predict the positive emotional samples in the original sample as positive emotional samples. This is also called TP (True Positive). The other is to predict the positive emotional samples in the original sample as negative emotional samples. This is also called FP (False Negative). The formula for calculating the recall rate is as follows:

9 $$recall=\frac{TP}{TP+FN}$$

The value ranges of precision and recall are both in [0,1]. Generally speaking, the higher the precision and recall are required to judge the performance of the model in this paper, the better. However, in actual situations, there may be other contradictions between P and R indicators, so F-Measure came into being as a comprehensive evaluation indicator for weighted balance adjustment of precision and recall. At this time, there will be two situations. The first one is to correctly predict the positive emotion sample (negative emotion) as the sample of the positive emotion sample (negative emotion). This is also called TP (True Positive). The other is to predict positive emotion samples (negative emotion) as negative emotion samples (positive emotion) samples. This is also called FP (False Positive). The comprehensive evaluation index (F-Measure) can be calculated as follows:

10 $$F=\frac{2\, \mathrm{x}\,p recision \mathrm\,{x\, recall}}{precision\,+\,recall}$$

### Experiment settings

The experiments in this paper use RSDD, eRisk2017 and eRisk2018 datasets. The introduction of RSDD and eRisk2017 dataset will not be repeated here. In particular, the training set of the eRisk2018 anorexia dataset contains 20 anorexic users and 132 control users, while the test set contains 41 anorexic users and 279 control users. Table 4 shows the statistics of the training dataset after word segmentation. The symbols that need to be marked are c, l, M and N. c represents the number of target categories, l represents the average length of posts, M represents the average number of posts by users, and N represents the size of the dataset. The size of the vocabulary is represented by |V|.

**Table 4:** Statistics after preprocessing of the training set of the three data sets

dataset	c	l	M	N	|V|
RSDD	2	148	969	38,823	966,881
eRisk2017	2	28	372	486	13,608
eRisk2018	2	41	373	152	17,400

Table 5 lists the values ​​of the hyperparameters used in the experiments in this chapter. This paper tries to gradually increase the maximum number of posts starting from 400 posts, but the performance of the verification dataset has not been significantly improved. So, we finally chose 400, 350, 350 as the maximum number of posts for the 3 datasets.

**Table 5:** Hyperparameter settings

Dataset	Max_m	Max_n	Embed_size	lr	Batch_size
RSDD	400	100	50	0.001	64
eRisk2017	350	30	100	0.001	64
eRisk2018	350	30	100	0.001	64

## Results and analysis

**Fig. 7:**
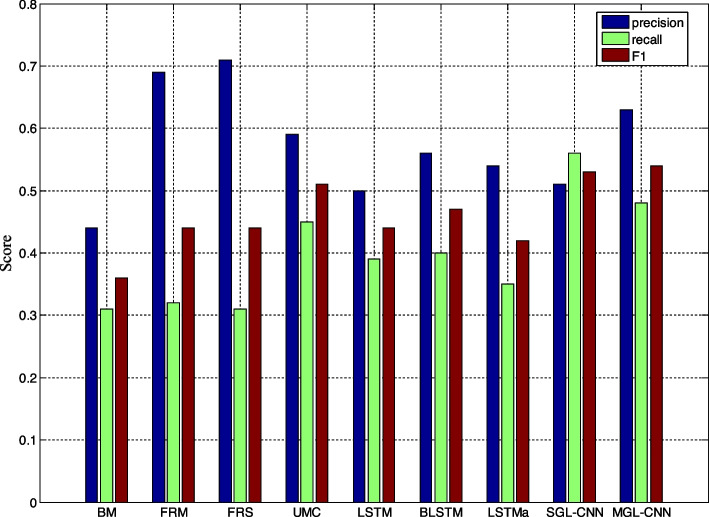
Comparison of graphical experimental results between the model proposed in this paper and the reference model on the eRisk2017 dataset

This sub-section mainly makes a comparative analysis of the final detection effect of the model proposed in this paper and the detection effect of the comparison model. Table 6 and Fig. 7 compare the performance of the 2 models proposed in this paper and other comparative models on the RSDD dataset. The results showed that the difference between the two models proposed in this paper and the reference model was statistically significant. We first compare with MNB and SVM classifiers using sparse and rich features. The results show that although MNB and SVM can achieve better results in accuracy, they perform poorly in terms of Recall and F1 indicators compared with CNN and LSTM-based models. For example, Feature-rich-MN and Feature-rich-SVM can reach 0.69 and 0.71 in terms of accuracy, but only 0.32 and 0.31 in Recall. In the mental disease detection task, if the Recall is higher, it means that more potential sick users can be detected, so as to avoid the problem of missed diagnosis. Therefore, for the special task of mental illness detection, more attention should be paid to Recall and F1.

**Table 6:** Comparison of experimental results between the model proposed in this paper and the reference model on the RSDD dataset

Method	precision	recall	F1
BoW-MNB	0.44	0.31	0.36
Feature-rich-MN	0.69	0.32	0.44
Feature-rich-SVM	0.71	0.31	0.44
User model-CNN	0.59	0.45	0.51
LSTM	0.50	0.39	0.44
Bi-LSTM	0.56	0.40	0.47
LSTM-attention	0.54	0.35	0.42
SGL-CNN	0.51	0.56	0.53
MGL-CNN	0.63	0.48	0.54

**Fig. 8:**
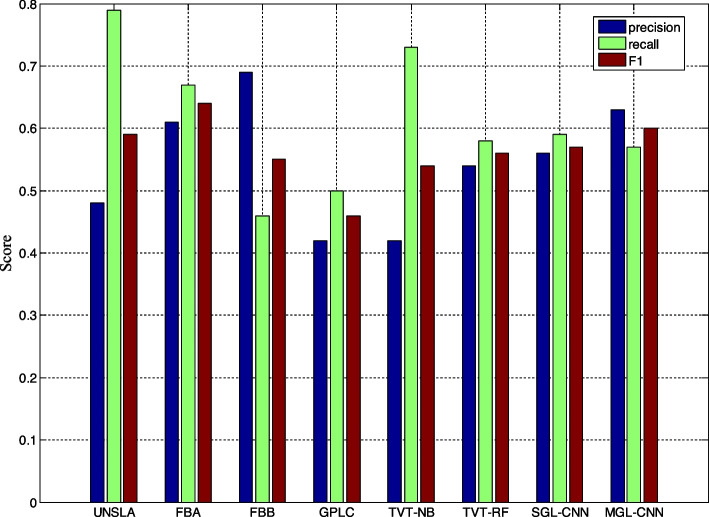
Comparison of graphical experimental results between the model proposed in this paper and the reference model on the RSDD dataset

Experimental results also show that the attention mechanism can help LSTM achieve good results in detection tasks. Compared with previous work, the proposed SCL-CNN model outperforms user-model-CNN in both Recall and F1. Table 7 and Fig. 8 compare the experimental results of the model proposed in this paper and the reference model on the early depression detection dataset. The results show that the performance of the model in this paper is close to the most advanced methods in terms of precision, recall and F1. Table 8 shows the experimental results of the proposed model and other state-of-the-art methods on the anorexia detection dataset. From the data value of the reference model, it can be known that the method with the highest accuracy rate is UNSLD, which achieves an effect of 0.91. But its recall is the worst, only 0.71. Compared with other reference models, the MGL-CNN model proposed in this paper has more balanced results on the three indicators, among which the F1 value is the highest, reaching 0.85. Compared with FHDO-BCSGE, which has the best accuracy index in the reference model, the work in this paper has a lower precision and F1 value, but the recall rate is 2.4% higher. In addition, compared with FHDO-BCSGE, the recall rate of MGL-CNN is also increased by 0.12%, and the F1 value is the same as it. The improvement of the recall rate means that more sick users can be detected in the anorexia detection task, which reduces the detection omission rate, and is more conducive to adjuvant treatment and providing help for more sick users.

**Table 7:** Comparison of experimental results between the model proposed in this paper and the reference model on the eRisk2017 dataset

Method	precision	recall	F1
UNSLA	0.48	0.79	0.59
FHDO-BCSGA	0.61	0.67	0.64
FHDO-BCSGB	0.69	0.46	0.55
GPLC	0.42	0.50	0.46
TVT-NB	0.42	0.73	0.54
TVT-RF	0.54	0.58	0.56
SGL-CNN	0.56	0.59	0.57
MGL-CNN	0.63	0.57	0.60

**Table 8:** Comparison of experimental results between the model proposed in this paper and the reference model on the eRisk2018 dataset

method	precision	recall	F1
FHDO-BCSGE	0.87	0.83	0.85
LIRB	0.79	0.73	0.76
UNSLD	0.91	0.71	0.79
UPFC	0.76	0.71	0.73
SGL-CNN	0.82	0.85	0.83
MGL-CNN	0.86	0.84	0.85

## Conclusion

With the continuous development of social media, there are now many studies on detection models for mental illnesses such as depression and anorexia. Mental illness is a broad public health topic. It is related to everyone's quality of life and family happiness index, as well as social and economic development and harmony. In the face of a large amount of user data, how to effectively mine the valuable content and implement the research on mental disease detection and improve its practicability is a big challenge. In this paper, two detection methods based on a hierarchical emotion detection model are proposed to identify patients with mental illness. Compared with the previous methods, the mental illness detection model proposed in this paper is more accurate and effective. The model proposed in this paper can effectively represent the user's overall emotional state by encoding the user's posts. In the experimental part, we validate our model on the large-scale RSDD and esk2017 depression detection dataset, erisk2018 anorexia detection dataset. Its results show that our proposed model significantly outperforms the existing best methods in terms of accuracy, recall and F1. In the future, we will try to conduct research on mental illness detection based on multi-modal data. For example, we effectively integrate information in three data formats: image, voice and text to better identify sick users.

## Data Availability

The experimental data used to support the findings of this study are available from the corresponding author upon request.
